# Medical and biomedical research productivity in Bahrain: an analysis of gender differences and patterns over two decades

**DOI:** 10.3389/frma.2026.1714436

**Published:** 2026-02-04

**Authors:** Randah R. Hamadeh, Enjy Khedr, Haitham Jahrami

**Affiliations:** 1 College of Medicine and Health Sciences, Arabian Gulf University, Manama, Bahrain; 2 Benha University, Benha, Egypt; 3 Government Hospitals, Manama, Bahrain

**Keywords:** biomedical, disparity, gender, medical, research output, research productivity, Scopus

## Abstract

**Introduction:**

Gender disparities in medical and biomedical research productivity remain a critical issue globally, yet data from Arab countries, including Bahrain, are limited. This study examines gender differences in medical and biomedical research productivity in Bahrain over a two-decade period (2005–2024), focusing on publication rates, authorship patterns, journal quality, and collaboration trends.

**Methods:**

A bibliometric analysis was conducted using the Scopus database, covering 5,445 publications from Bahraini institutions in the medical and biomedical fields. Data were filtered to include only articles and reviews. Books, conference papers, and retracted works were excluded. The variables included authorship positions (first, last, corresponding), journal quartiles, SCImago Journal Rank (SJR), and collaboration types (institutional, national, regional, international). Gender was determined based on the authors' names and institutional and professional profiles. Descriptive statistics, Student's *t*-tests, and chi-square tests were used to compare sex differences (*p* < 0.05).

**Results:**

The number of medical and biomedical publications in Bahrain increased fivefold from 2005 to 2024, comprising 80.1% of the articles and 12.8% of the reviews. Arabian Gulf University led institutional output (30.2% of total publications). Local journals accounted for 54.0% of the publications, with limited international visibility. Males from Bahrain dominated authorship (59.3% first, 68.3% last, 65.6% corresponding), whereas females comprised 36.0% of Bahraini authors on average. Male first authors from Bahrain were more prevalent in Q1 journals (58.3% vs. 41.7%) and international collaborations (40.7%). The female corresponding authors included more female Bahraini coauthors (mean 78.19% vs. 15.99% for males).

**Conclusion:**

Gender disparities in Bahrain's medical and biomedical research output underscore the need for targeted interventions to support female researchers, particularly in securing senior authorship and publishing in high-impact journals. Enhancing international collaboration and equitable resource allocation could further strengthen Bahrain's research landscape.

## 1 Introduction

Academic publications have become highly valued by universities, as they are among the criteria considered in world university rankings, and universities take pride in having the names of their faculty members listed on global data platforms such as the World Ranking of the Top 2% Scientists (Stanford University Elsevier, 2025). Moreover, research productivity is a significant contributing factor to academic recruitment, the promotion of current academic staff, the maintenance of current jobs, and recognition among scientists (Lerchenmuller et al., 2018). Thus, measuring research productivity has become paramount as academic medicine has become increasingly competitive. There are several publication-based metrics of scholarly productivity, including the number of published research papers, authorship order, publication sources, and article citations (Ranjan et al., 2016).

Although women are increasingly represented in some academic disciplines (Ortega et al., 2024), female researchers remain underrepresented in the production of scientific literature (Macaluso et al., 2016; Giannos et al., 2023; Gutiérrez Torres et al., 2025), particularly in lower-middle-income and low-income countries (Totis et al., 2025). A study revealed that Chilean female education researchers published, on average, 20.8% fewer articles than their male counterparts did. There were no gender differences in research leadership, such as first authorship, but male researchers had more international research collaborations, whereas women researchers engaged more in national collaboration (Hiller et al., 2020). However, women's authorship in high-impact orthopedic journals has increased, and the gender gap has narrowed (Grant et al., 2024).

There has been more focus on gender disparities in scientific publications. Gender differences were reported in terms of productivity, collaboration efforts, and citations (Mayer and Rathmann, 2018). Women tend to publish fewer articles than their male counterparts do, often in less prominent journals, and may opt for less prestigious outlets, such as book chapters. This pattern could disadvantage women in advancing academically, as peer-reviewed journal publications are vital for career advancement, obtaining tenure, securing research funding, and gaining recognition within the scientific community, and attaining leadership roles (Mayer and Rathmann, 2018; Gutiérrez Torres et al., 2025). Moreover, women's articles receive fewer citations than their male peers do, which suggests a lower influence on their publications (Lerchenmuller et al., 2018). Several individual factors influence this gender gap, including career age, rank, marital status, motivation, personality, talent, and specialization. In addition, several organizational factors are involved, including department size and setup, research group size, university prestige, research focus, funding, collaboration, and coauthorship.

The persistent trend of men publishing more scientific papers than women throughout their careers has been referred to as the “productivity puzzle” (Huang et al., 2020). The “productivity puzzle,” which captures the enduring pattern of men publishing more than women throughout their careers, captures the heart of this gender gap. There are several possible reasons for this discrepancy, including variations in family obligations and job interruptions, issues with resource allocation, peer review procedures, teamwork, role expectations, academic standing, specialty, and workplace culture (Huang et al., 2020). However, when research productivity is examined after controlling for having children, gender remains a major predictor of research productivity (Stack, 2004). This gender gap remains evident even after accounting for career length and is observed across most countries and disciplines. Various individual and organizational factors have been proposed to elucidate this phenomenon, including marital status and the presence of children (Stack, 2004) career interruptions, and the disproportionate family responsibilities shouldered by women (Lerchenmuller et al., 2018; Mayer and Rathmann, 2018), differences in academic rank and specialization (Mairesse and Pezzoni, 2015), personality and motivational traits (Huang et al., 2020; Mayer and Rathmann, 2018), unequal access to funding, larger research groups, and prestigious collaboration networks (Macaluso et al., 2016; Lerchenmuller et al., 2018), as well as systemic biases in the recognition of women's contributions—historically termed the “Matilda effect” (Rossiter, 1993). While the relative impact of each factor varies by country, discipline, and institutional context, their cumulative effect consistently disadvantages female researchers in terms of publication metrics and career advancement.

A review of health research in the Eastern Mediterranean region revealed that research and development (R&D) funding is low in Bahrain and the other Gulf States, except for Qatar (Ismail et al., 2013). The biomedical research output of Bahrain and other Arab countries lagged behind that of Turkey, Iran, and Israel (Benamer and Bakoush, 2009). However, a review conducted in Saudi Arabia revealed a 14.1% increase in health research publications from 2008 to 2017 (Ul Haq et al., 2020), and biomedical research in Qatar increased almost 24 times from 2000 to 2012 (Zeeneldin and Taha, 2014). Recent mapping of Middle East research from 2003 to 2023 revealed that 20.9% of Bahraini publications are in the health sciences, 49.6% in the physical sciences, 20.6% in the social sciences, and 8.9% in the life sciences. Bahrain was reported to have 56.8% international research collaboration, the lowest percentage among Gulf Cooperation Council (GCC) countries after Kuwait (Dardas et al., 2023). Between 2007 and 2016, Bahrain published 608 medical articles and contributed 0.9% to the Arab world's medical research output (El Rassi et al., 2018).

Research on gender differences in medical and biomedical research productivity in Arab countries is scarce. A 10-year study examining the research productivity of medical faculty at the American University of Beirut reported that women researchers had a lower mean number of publications (20.8) compared to men (30.2), and they were underrepresented in senior research authorship (Rachid et al., 2021). Similar findings were reported from two reviews of Qatar and GCC countries that examined mental health research productivity by gender, where men had more research output and had more senior authorship positions (Albahari and Bashir, 2020b,a). Studies on gender disparities in research have not yet been conducted in Bahrain, and this study, examining medical and biomedical research, would hopefully identify gaps and suggest ways to narrow them.

Bahrain has 19 universities and higher education institutions, of which only four include medical and biomedical studies. They are Arabian Gulf University (AGU), the Royal College of Surgeons Ireland-Medical University of Bahrain (RCSI-MUB), the University of Bahrain (UOB), and Ahlia University. Bahrain has public health institutions (PHIs) that comprise the Ministry of Health (MOH), which includes primary healthcare centers, the Public Health Directorate, government hospitals (Salmaniya Medical Complex (SMC), Psychiatric Hospital, Jidhafs Maternity Hospital, and Geriatric Hospital), and the Royal Medical Services (RMS). The latter includes the Military Hospital (BDF), the Mohammed bin Khalifa bin Salman Al Khalifa Cardiac Center (MKCC), and King Hamad University Hospital (KHUH), which houses the Bahrain Oncology Center (BOC). Government hospitals (GHs) became autonomous in 2020, and before that, they were part of the MOH. The MKCC was established in 1992, and the BOC was established in 2019. In addition to the public sector, Bahrain has several private hospitals and clinics.

The study's objectives were to determine the gender gap in the rates of medical and biomedical publications in Bahrain from 2005 to 2024, describe the trend of publication rates by gender, compare the authorship patterns among men and women (first, last, and corresponding authors, and the five other coauthors), compute the proportions of medical and biomedical publications by gender of first, last and corresponding authors, determine the differences in the quality of journals by gender, determine the number of citations of articles by gender, compute the trend of the average proportion of female authors, compute the average percentage of Bahraini institutions involved out of the total institutions, and identify the leading Bahraini organizations contributing to the country's medical and biomedical research output.

## 2 Materials and methods

The Scopus database was utilized for a bibliometric analysis of medical and biomedical publications affiliated with Bahrain, covering the 20-year period from January 1, 2005, to December 31, 2024. This timeframe was chosen to provide a balanced, symmetrical, and easily interpretable long-term perspective, which is a common approach in bibliometric studies examining research productivity and gender equity. Extending the analysis to earlier years would have included periods with very low publication output (fewer than 10–15 papers annually before 2005), resulting in statistically unstable gender proportions and potentially misleading trends.

The data retrieval process was designed to ensure comprehensive coverage while maintaining relevance. An initial broad search in Scopus for Bahrain-affiliated publications within the specified medical and biomedical subject areas (including Biochemistry, Genetics and Molecular Biology; Dentistry; Health Professions; Immunology and Microbiology; Medicine; Multidisciplinary; Neuroscience; Nursing; Pharmacology, Toxicology and Pharmaceutics; and Veterinary) yielded 6,128 records. After restricting the results to preferred document types—such as articles, editorials, letters, notes, reviews, and short surveys—while excluding books, book chapters, conference papers, corrections, errata, retracted articles, and items in press, the dataset was reduced to 5,766 records.

To capture additional relevant publications, targeted searches were conducted for major academic institutions (AGU, RCSI-MUB, UOB) and health institutions (MOH, KHUH, and BDF) in Bahrain. These searches identified 4,991 records. The country-level and institutional datasets were then merged, producing a combined total of 10,757 records.

**Figure 1:**
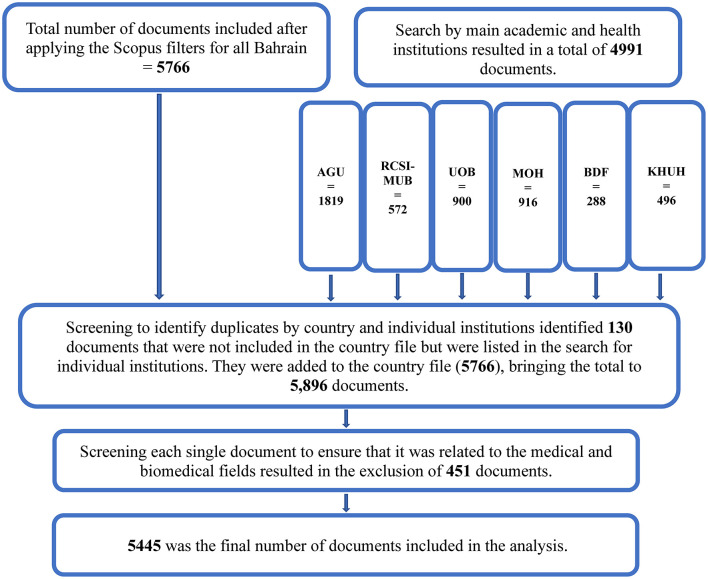
Retrieved documents from Bahraini organizations for the period between 2005 and 2024. MOH, Ministry of Health; RCSI-MUB, Royal College of Surgeons Ireland-Medical University of Bahrain; UOB, University of Bahrain.

Duplicates within the merged dataset were identified and removed using Excel's duplicate removal function based on matching DOIs, titles, and links, eliminating 4,861 records and leaving 5,896 unique papers. This step revealed that the institutional searches added only 130 unique records not captured in the initial country-based search. Finally, manual screening of all records was performed to confirm strict relevance to the medical and biomedical fields, leading to the exclusion of 451 papers. The resulting final dataset comprised 5,445 publications, which formed the basis of the analysis (Figure 1).

In addition to the variables extracted from Scopus, we included the following variables in the Master file: authors from Bahrain (name, sex and affiliation of the first author, last author, corresponding author, and the first five coauthors), journal quartile and SCImago Journal Rank (SJR), total number of authors, number of authors from Bahrain (male, female, and total), the percentage of authors from Bahrain, percentage of females among authors from Bahrain from total authors from Bahrain, total number of institutions, number of institutions from Bahrain, percentage of institutions from Bahrain, and type of collaboration (none, institutional, regional, and international). Major institutions were added, including AGU, RCSI-MUB, UOB, MOH, SMC, Psychiatric Hospital, BDF, KHUH, and GH. The rest were grouped into research centers and other institutions, which included private hospitals and clinics, and other organizations.

The sex of Arab authors, who were the majority, was known from their first names. In the rare cases of the neutral names of Arab authors and the uncertainty of the sex of non-Arab authors, full names were searched on the websites of their affiliated institutions, Scopus Profiles, Google Scholar, and LinkedIn. The first Bahraini affiliation documented in the article was used for authors with multiple affiliations. The computation of the number of authors and institutions was restricted to those 15 and below. The types of collaboration, i.e., single author (none), institutional (authors from the same institution), national (authors from different institutions within the same country), regional (authors from Arab countries), and international (collaborations with authors from other countries), were identified. The threshold of 15 authors was chosen as a pragmatic and widely adopted cut-off in large-scale bibliometric studies because it excludes the small proportion of highly multi-authored consortium papers (typically large epidemiological registries, genome-wide association studies, or international clinical trials) that disproportionately inflate the mean number of authors and institutions while contributing minimal additional information on individual-level authorship patterns. In our dataset, 463 publications (8.5% of the total) had >15 authors; these were excluded solely from the calculation of the average number of authors and institutions per paper to prevent distortion of descriptive statistics, as is standard practice (Huang et al., 2020; Lariviere and Costas, 2016).

A separate sensitivity analysis was conducted on these 463 excluded papers in the computation of the number of authors: they were published predominantly in Q1/Q2 international journals (78.4%), had higher citation rates, and showed a slightly larger gender gap in senior positions (last author 71.9% male vs. 68.3% in the main sample; corresponding author 69.2% male vs. 65.6%. Among the publications with more than 15 authors, there were only three first authors (2 male, 1 female), three last authors (2 male, 1 female), and three corresponding male authors from Bahrain, while the rest were coauthors. However, because these papers represent only 8.5% of the total dataset and the direction of the gap remains unchanged, their exclusion does not alter any of the study's conclusions regarding overall gender disparities.

The percentages of Bahraini authors, female Bahraini authors, and Bahraini institutions were computed. If the partnership was international, any regional collaboration within it was disregarded. We assigned the journal quartile and SJR where applicable, using the official SJR database for the relevant publication year. If the journal had more than one specialty, the quartile related to the document specialty was used, and if more than one specialty applied, the highest quartile was selected. The data were entered and analyzed via SPSS. Means and standard deviations of continuous variables were compared between sexes via Student's independent t-test. Categorical variables were compared by sex via the chi-square test. Furthermore, we observed that several key variables-specifically citation counts, the number of authors/institutions, and annual proportions of female authorship-exhibited strongly right-skewed distributions. This was evidenced by standard deviations significantly exceeding the means and a notable departure from normality, as assessed through visual inspection of histograms and Shapiro-Wilk tests. Consequently, we supplemented the parametric analyses with non-parametric methods where appropriate. The Kruskal-Wallis test was utilized to compare the distribution of the annual proportion of Bahraini female authors among all Bahraini authors over the 20-year study period, given the marked skewness, heteroscedasticity, and ordinal-like characteristics of this variable over time. The choice of the Kruskal-Wallis test over repeated-measures ANOVA was based on the non-normal distribution and unequal variances across years. The results of the Kruskal-Wallis test are presented, along with the median and interquartile range (IQR) to provide a more comprehensive description of central tendency and dispersion in the context of skewness. A *p* < 0.05 was considered statistically significant.

## 3 Results

### 3.1 Overall description

#### 3.1.1 Temporal trends and document types

**Figure 2:**
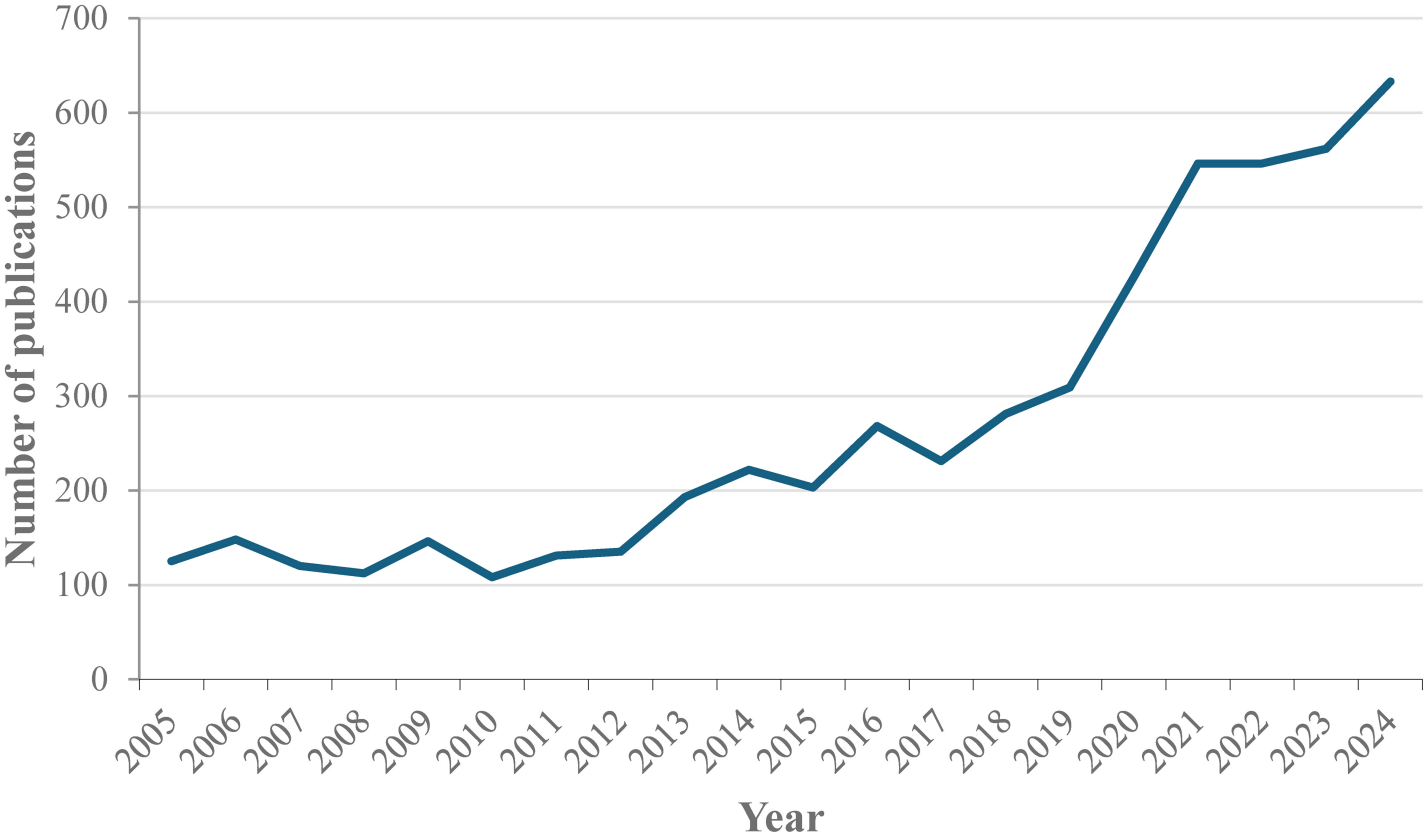
Distribution of publications by year.

The dataset comprises 5,445 scientific publications from Bahrain, spanning the period from 2005 to 2024. Figure 2 shows the temporal distribution of publications during that period. There was a marked increase in publication output over time, with a fivefold increase in publications between 2005 and 2024 and notable spikes in 2020 and 2021. The document types were as follows: article (4,363, 80.1%), review (694, 12.8%), letter (135, 2.5%), editorial (125, 2.3%), note (78, 1.4%), and short survey (50, 0.9%).

#### 3.1.2 Quantitative descriptive characteristics of the publications

Table 1 presents the descriptive statistics for key quantitative variables related to the characteristics of scientific output. There were 19,535 authors, of whom 9,791 were from Bahrain, over the total period. There were 463 manuscripts with more than 15 authors, including 3 from Bahrain. The mean number of authors was 5.1 ± 3.2, and that of authors from Bahrain was 2.3 ± 1.9.

**Table 1:** Descriptive statistics of publication characteristics of documents (*n* = 5,445).

**Variable**	**Mean**	**SD**	**Minimum**	**Maximum**
Total number of authors (*n =* 19,535)^*^	5.1	3.2	1.0	15.0
Number of authors from Bahrain (*n =* 9,791)^$^	2.3	1.9	1.0	15.0
Proportion of authors from Bahrain (%)^*^$	60.3	38.6	3.1	100.0
Number of institutions involved (*n =* 11,196)^**^	3.0	2.5	1.0	15.0
Number of Bahraini institutions (*n =* 4,891)	1.2	0.5	1.0	6.0
Proportion of Bahraini institutions (%)^**^	61.8	35.8	6.7	100.0

^*^The number of authors above 15 (463) was excluded. ^*$*^The number of authors from Bahrain above 15 (3) was excluded. ^**^The number of institutions above 15 (390) was excluded.

#### 3.1.3 Journal features

There were 1,856 journals, with English dominating the publication language, accounting for 99.9% of all publications (*n* = 5,440), with minimal representation of other languages, including Arabic, French, German, and Turkish (collectively <0.1%). In 183 (3.4%) documents, the journals had neither an assigned quartile nor an SJR. Of the 5,262 documents that were indexed, the average SJR was 1.0 ± 1.9, with publications ranging from low-impact journals (SJR = 0.1) to high-impact journals (SJR = 24.2). Publications were distributed across all journal quartiles, with a notable concentration in Q1 (31.7%) and Q3 (24.2%) journals. Q2 journals accounted for 22.1%, and Q4 journals accounted for 3.3%. The average overall number of citations per document was 39.5 ± 374.4, ranging from 0 to 13,221.

Almost two-thirds of the journals were local or regional journals. The three local journals (Bahrain Medical Bulletin, Journal of the Bahrain Medical Society, and the Arab Gulf Journal of Scientific Research) accounted for 54.0% of all the journals in which manuscripts were published during the period. The five regional journals (Saudi Medical Journal, Eastern Mediterranean Health Journal, Oman Medical Journal, Annals of Saudi Medicine, and Sultan Qaboos University Medical Journal) accounted for 9.0% (Table 2).

**Table 2:** Journals with the highest proportion of publications.

**Rank**	**Journal**	**Frequency**	**%**
1	Bahrain Medical Bulletin	808	43.5
2	Journal of the Bahrain Medical Society	148	8.0
3	Saudi Medical Journal	66	3.6
4	The Lancet	58	3.1
5	PLoS ONE	50	2.7
6	Arab Gulf Journal of Scientific Research	46	2.5
7	Cochrane Database of Systematic Reviews	46	2.5
8	BMJ Case Reports	42	2.3
9	Nutrients	41	2.2
10	Scientific Reports	37	2.0
11	Eastern Mediterranean Health Journal	36	1.9
12	International Journal of Surgery Case Reports	28	1.5
13	International Journal of Molecular Sciences	26	1.4
14	British Journal of General Practice	26	1.4
15	Frontiers in Endocrinology	25	1.35
16	Angiology	25	1.4
17	Oman Medical Journal	24	1.3
18	Diabetes Research and Clinical Practice	23	1.2
19	Journal of Infection and Public Health	21	1.1
20	Annals of Saudi Medicine	21	1.1
21	World Journal of Clinical Pediatrics	21	1.1
22	Sultan Qaboos University Medical Journal	20	1.1
23	BMJ Open	20	1.1
24	Radiology Case Reports	20	1.1
25	Others	178	9.6
	Total	1,856	100.0

#### 3.1.4 Institutional affiliations, author demographics, and collaboration patterns

A substantial proportion of publications (41.4%, *n* = 2,254) involved institutions exclusively from Bahrain. Collaboration between Bahraini and international institutions accounted for 40.7%, whereas collaboration with regional institutions reached 17.9%. The exclusive Bahraini involvement was institutional (23.8%), followed by local collaboration with other Bahraini institutions (10.2%) and the participation of 7.5% of single authors.

**Figure 3:**
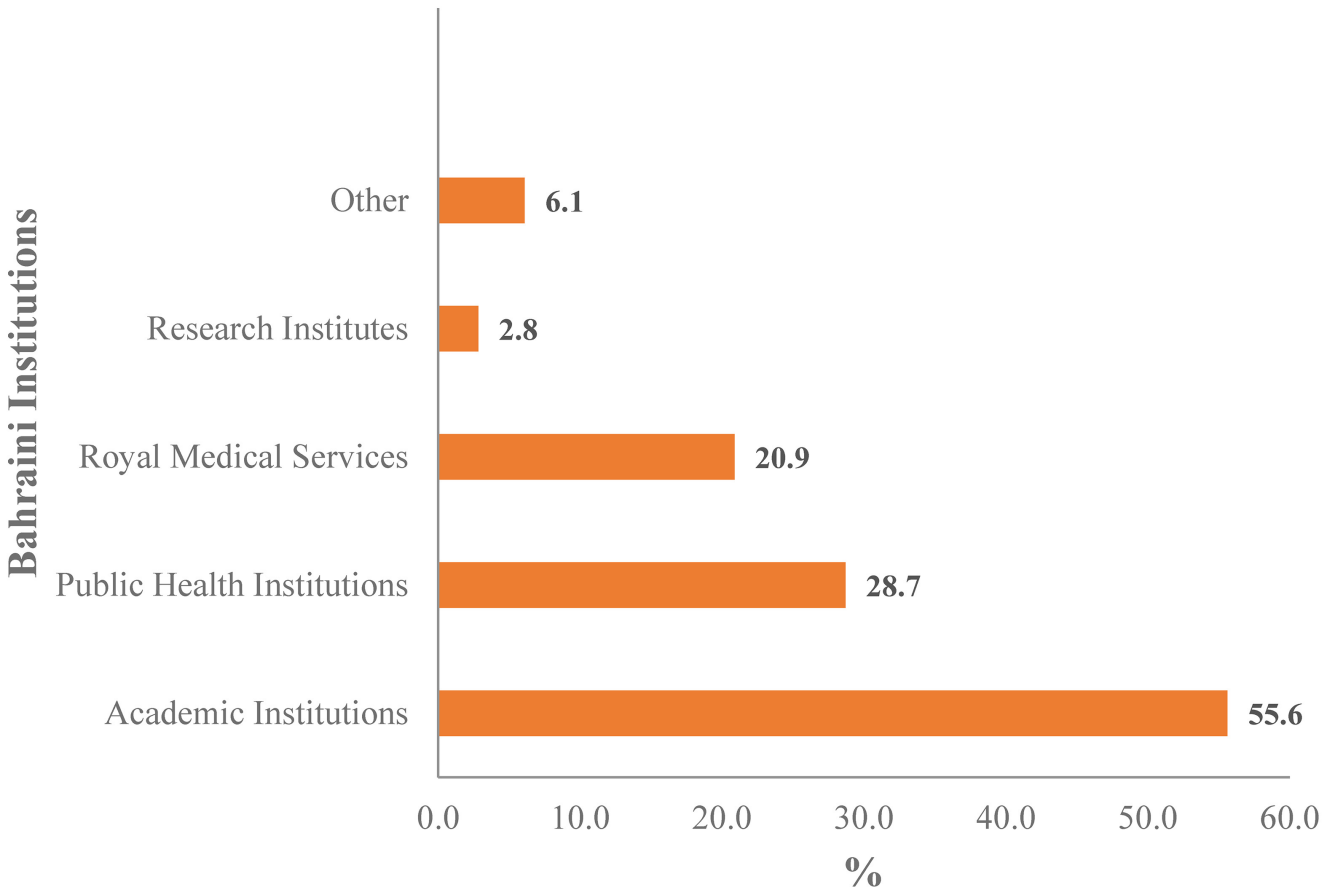
Publications by Bahraini institutions.

Academic institutions contributed 55.6% of the publications, followed by PHIs (28.7%), RMS (20.9%), research institutes (2.8%), and other private clinics, centers, and hospitals (6.1%) (Figure 3). Over half (54.2%) of the publications of academic institutions and 30.2% of the total publications had an AGU author. AGU was the leading institution across all authorship positions, accounting for approximately 24.8% to 28.8% of the authors in each category (Table 3). It was followed by SMC (17.2%−19.6%), KHUH (9.6%−11.2%), RCSI-MUB (10.0%−11.1%), and BDF (10.3%−10.9%).

**Table 3:** Top 5 institutional affiliations by author position in all publications with Bahraini authors.

**Institution**	**First author**	**Last author**	**Corresponding author**
Arabian Gulf University	792 25.3	693 27.4	884 29.3
Salmaniya Medical Complex, Psychiatric Hospital, and Governmental Hospitals	670 21.4	531 21.0	574 19.0
Military Hospital and Mohammed bin Khalifa bin Salman Al Khalifa Cardiac Center	382 12.2	301 11.9	350 11.6
Royal College of Surgeons Ireland-Medical University of Bahrain	336 10.7	252 10.0	335 11.1
King Hamad University Hospital and Bahrain Oncology Center	312 10.0	289 11.4	298 9.9
University of Bahrain	208 6.7	142 5.6	186 6.2
Primary healthcare, Public Health, and other Ministry of Health departments	200 6.4	147 5.8	179 5.9
Other	230 7.3	174 6.9	209 6.9
Total	3,130 100.0	2,529 100.0	3,015 100.0

Publications that had 15 authors or fewer (4,982) typically involved an average of 5.1 authors, with authors from Bahrain representing approximately 81% of all authors per publication. Three thousand one hundred thirty publications (57.5%) involved the first author from Bahrain, 2,529 (46.4%) involved the last author, and 3,015 (55.4%) involved the corresponding author. Bahraini authors constituted 87 to 97.8% of the first authors who published in local journals, the last authors constituted 70.0 to 75.5%, and the corresponding authors constituted 84.8 to 90.8% (Supplementary Table 1). The predominant type of collaboration was institutional collaboration, representing 40.7% of all publications (*n* = 2,214), followed by regional collaboration (23.7%, *n* = 1,292) and institutional collaboration (17.9%, *n* = 974). Local collaboration accounted for 10.2% (*n* = 555), whereas 7.5% of the publications (*n* = 406) were single-authored and involved no collaboration.

### 3.2 Gender gap

#### 3.2.1 Authorship

Of the 9,791 authors from Bahrain, determining the gender of less than 1% was challenging. However, gender could be inferred from multiple publications of the same author and affiliation.

Compared with females, male authors from Bahrain (4.0–4.6) in all authorship positions had a greater number of coauthors (3.7–3.9). The overall mean proportion (36.0 ± 40.5) of female authors among all those from Bahrain was lower than that of male authors (Table 4). The mean number of male first authors from Bahrain was 3.1 ± 2.2, and that of females was 3.1 ± 2.1. In contrast, the mean number of last authors was 3.4 ± 2.2 for males and 3.5 ± 1.9 for females. The mean numbers of male and female corresponding authors from Bahrain were similar, at 3.1 ± 2.2 and 3.1 ± 2.0, respectively. The female first authors tended to include female coauthors (average mean 71.2% ± 28.8), whereas the male first authors included an average of 19.4% ± 25.5%. For the corresponding authors, the values were 73.1% ± 28.3% and 20.7% ± 26.0%, respectively, and for the last author, the values were 67.8% ± 27.7% and 23.4% ± 26.3%, respectively (data not presented).

**Table 4:** Changes in the proportion of Bahraini female authorship from total Bahrainis across the study period.

**Year**	**Number of documents**	**Mean ±SD**	**Median (IQR)**	***p*-value**
2005	125	26.1 ± 37.5	0.0 (0.0–50.0)	**<0.001**
2006	148	30.0 ± 37.7	0.0 (0.0–50.0)	
2007	120	32.1 ± 37.7	20.0 (0.0–50.0)	
2008	112	29.1 ± 37.9	0.0 (0.0–50.0)	
2009	146	29.4 ± 39.9	0.0 (0.0–54.2)	
2010	108	25.7 ± 34.1	0.0 (0.0–50.0)	
2011	131	33.6 ± 38.7	16.7 (0.0–60.0)	
2012	135	30.6 ± 38.1	0.0 (0.0–50.0)	
2013	193	34.0 ± 40.0	0.0 (0.0–66.7)	
2014	222	34.2 ± 40.0	11.3 (0.0–66.7)	
2015	203	33.7 ± 40.0	0.0 (0.0–60.0)	
2016	268	35.5 ± 39.7	25.0 (0.0–66.7)	
2017	231	35.1 ± 40.0	25.0 (0.0–66.7)	
2018	281	40.9 ± 41.0	33.3 (0.0–100.0)	
2019	309	34.6 ± 40.0	25.0 (0.0–66.7)	
2020	426	37.0 ± 39.9	25.0 (0.0–66.7)	
2021	545	37.2 ± 40.2	25.0 (0.0–66.7)	
2022	546	40.1 ± 42.5	25.0 (0.0–100.0)	
2023	560	37.3 ± 41.8	20.0 (0.0–83.0)	
2024	632	40.5 ± 42.6	33.3 (0.0–100.0)	
Total	5,441	36.0 ± 40.5	20.0 (0.0–66.7)	

Four documents were excluded (one in 2024, two in 2023, and one in 2021) because the number of authors exceeded 15. IQR, Interquartile range. The Kruskal–Wallis test was used. Statistically significant *p*-values are formatted in bold.

**Figure 4:**
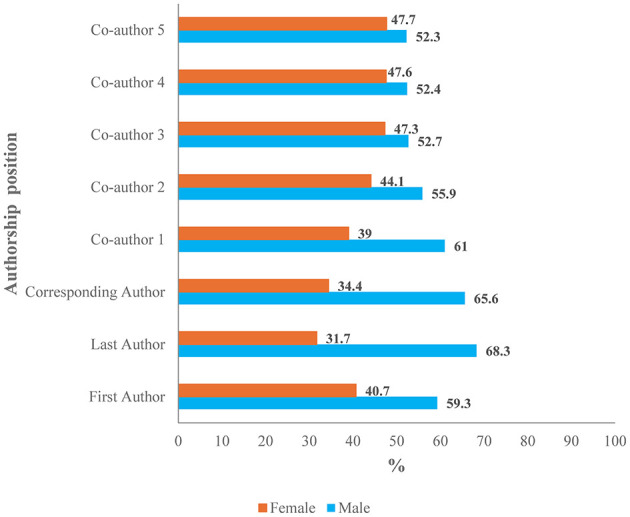
Gender distribution by author position.

The gender gap was statistically significant throughout the years. The lowest percentage (25.7% ± 34.1%) was recorded in 2010, and the highest percentage (40.9% ± 41.0%) was recorded in 2018 (Table 4). Across all authorship positions, male authors consistently outnumbered female authors (Figure 4). For the first authors, males comprised 59.3% (*n* = 1,857) of the sample, whereas females accounted for 40.7% (*n* = 1,273) of the sample. This gender disparity was more pronounced for last authors (males: 68.3%, *n* = 1,726; females: 31.7%, *n* = 802) and corresponding authors (males: 65.6%, *n* = 1,980; females: 34.4%, *n* = 1,038). The proportions of male first to fifth coauthors from Bahrain were slightly greater than those of female coauthors (Figure 4). The sex ratios of the first author, last author, and corresponding author from Bahrain were 1.5, 2.2, and 1.9, respectively.

As anticipated in bibliometric datasets, the distribution of several variables-including the annual proportion of female Bahraini authors, the total number of authors, and citation counts-exhibited a pronounced right skew, with standard deviations significantly exceeding the means. Mean ± standard deviation values are presented throughout the manuscript to enable comparison with prior studies in the field; however, readers are advised to interpret these descriptive statistics with caution. The observed gender differences in authorship patterns remained consistent and statistically significant when non-parametric methods were employed, as detailed in Table 4.

#### 3.2.2 Journal features: journal quartile, top leading journals

Male first authors had higher counts (353 vs. 252) and a higher percentage (58.3% vs. 41.7%) in Q1 journals, suggesting greater representation in top-tier publications. Similarly, males outnumbered females in the last and corresponding authors (Table 5). Male and female first and corresponding authors from Bahrain had equal mean journal SJR (0.5 and 0.6), whereas males had a slightly higher mean journal SJR than females did for last authors (0.6 ± 0.6 vs. 0.4 ± 0.4). The citations of journals in which a Bahraini male was the last author outnumbered those of females (8.5 +16.6 vs. 5.5 +14.2), and the corresponding author (8.5 +20.2 vs. 5.3 +11.6) was similar to the first author (7.4 ±19.5 vs. 7.7 ±50.6.

**Table 5:** Distribution of journal quartiles according to the gender of Bahraini first, last, and corresponding authors.

**Journal**	**First author**				**Last author**				**Corresponding author**			
	**Male**		**Female**		**Male**		**Female**		**Male**		**Female**	
	***n***	**%**	***n***	**%**	***n***	**%**	***n***	**%**	***n***	**%**	***n***	**%**
Q1 (*n =* 1,669)	353	19.6	252	20.4	349	20.7	128	16.7	385	20.0	202	20.2
Q2 (*n =* 1,202)	362	20.1	186	15.1	363	21.6	120	15.6	437	22.7	130	13.0
Q3 (*n =* 1,271)	526	29.2	377	30.5	466	27.7	227	29.6	547	28.5	290	28.9
Q4 (*n =* 1,120)	558	31.0	420	34.0	505	30.0	293	38.2	552	28.7	380	37.9
**Total**	1,799	100.0	1,235	100.0	1,683	100.0	768	100.0	1,921	100.0	1,002	100.0
***p*** **-value**	**0.005**				**<0.001**				**<0.001**			

Chi-square test was used. Statistically significant *p*-values are formatted in bold.

There were gender differences among authors from Bahrain in the journals most frequently chosen for publication, as indicated by the first, last, and corresponding authors (Supplementary Table 2). This finding was particularly pronounced in the local journal Bahrain Medical Bulletin, where 30.9% of the females were first authors, 30.7% were last authors, and 34.7% were corresponding authors, whereas 21.4%, 21.3%, and 18.9% were males, respectively (*p* < 0.001).

#### 3.2.3 Document type

There were statistically significant differences by document type and gender across authorships (Table 6). Slightly greater proportions of females from Bahrain than males were first authors (83.3%), last authors (87.9%), and corresponding authors (81.3%) in the original articles (78.7%, 85.3%, and 81.3%, respectively). The proportions of males were greater than those of females for almost all the other types, except for notes and short surveys (Table 6).

**Table 6:** Distribution of document types according to the gender of first, last, and corresponding authors from Bahrain.

**Document**	**First author**				**Last author**				**Corresponding author**			
	**Male**		**Female**		**Male**		**Female**		**Male**		**Female**	
	***n***	**%**	***n***	**%**	***n***	**%**	***n***	**%**	***n***	**%**	***n***	**%**
Article (*n =* 4,365)	1,463	78.7	1,060	83.3	1,461	85.3	705	87.9	1,611	81.3	876	84.4
Editorial (*n =* 125)	75	4.0	22	1.7	38	2.2	4	0.5	75	3.8	16	1.5
Letter (*n =* 135)	61	3.3	27	2.1	29	1.7	19	2.4	52	2.6	18	1.7
Note (*n =* 78)	40	2.2	25	2.0	14	0.8	10	1.2	38	1.9	21	2.0
Review (*n =* 694)	198	10.7	120	9.4	157	9.2	62	7.7	185	9.3	90	8.7
Short survey (*n =* 50)	21	1.1	19	1.5	14	0.8	2	0.2	20	1.0	17	1.6
**Total**	1,858	100	1,273	100	1,713	100	802	100	1,981	100	1,038	100
***p-*** **value**	**0.001**				**0.006**				**0.004**			

Chi-square test was used. Statistically significant *p*-values are formatted in bold.

#### 3.2.4 Collaboration

Among the total institutions, a greater percentage of female authors were from Bahraini institutions (84.5%−90.2%), whereas males represented a lower percentage (79.4%−80.3%) across all authorship positions (not presented). Males collaborated with more institutions (mean 1.9 ± 2.1) than females did (mean 1.7 ± 1.8) across all authorship positions (data not presented). Compared with female first authors, male first authors collaborated with more institutions (mean, 1.8 vs. 1.5) and Bahraini institutions (mean, 1.9 vs. 1.7). A greater percentage of male first authors than female first authors had regional and international collaborations (31.7% vs. 26.2%). In contrast, female first authors had higher counts of institutional collaborations (707 vs. 561). Last and corresponding authors from Bahrain had higher percentages of regional (15.0% vs. 8.5%) and international collaboration (15.4% vs. 6.6%) (Table 7).

**Table 7:** Collaboration type distribution by sex of author from Bahrain.

**Collaboration type**	**None**		**Institutional**		**Local**		**Regional**		**International**		**Total**	
**First author**												
	***n***	**%**	***n***	**%**	***n***	**%**	***n***	**%**	***n***	**%**	***n***	**%**
Female	163	12.8	561	44.0	216	17.0	123	9.7	211	16.6	1,274	100
Male	241	13.0	707	38.1	320	17.2	207	11.1	382	20.6	1,857	100
***p-*** **value**	**0.005**											
**Last author**												
	***n***	**%**	***n***	**%**	***n***	**%**	***n***	**%**	***n***	**%**	***n***	**%**
Female	0	0.0	472	58.9	194	24.2	68	8.5	68	8.5	802	100
Male	0	0.0	793	46.4	344	20.1	257	15.0	315	18.4	1,709	100
***p-*** **value**	**<0.001**											
**Corresponding author**												
	***n***	**%**	***n***	**%**	***n***	**%**	***n***	**%**	***n***	**%**	***n***	**%**
Female	160	15.4	484	46.6	169	16.3	68	6.6	157	15.1	1,038	100
Male	227	11.5	732	37.0	356	18.0	304	15.4	361	18.2	1,980	100
***p-*** **value**	**<0.001**											

Chi-square test was used. Statistically significant *p*-values are formatted in bold.

## 4 Discussion

Our study of the gender disparity in research productivity in Bahrain is highly important. This is the first study to examine the country's medical and biomedical research output, compare research productivity by gender, and analyze trends over a 20-year period. This study helps address the unique challenges faced by female scholars in Bahrain and the Arab region, reporting on the magnitude of the gender gap over the years. This study aims to contribute to ongoing efforts to promote gender equity in academia, ultimately benefiting the academic community and enhancing research output in the region.

Our study confirms the continuous increase in the research output of biomedical publications in Bahrain (Abu-Dawas et al., 2015; Bredan et al., 2011; Al-Farsi et al., 2021) with a notable increase during the COVID-19 pandemic, similar to what has been reported (Else, 2020; Sloane and Zimmerman, 2021). This reveals a persistent gender disparity in the productivity of medical and biomedical research in Bahrain over the past two decades, with male authors consistently outnumbering females across key authorship positions. This pattern aligns with the global “productivity puzzle,” where males tend to publish more throughout their careers (Huang et al., 2020). The proportions of female authors as first authors, corresponding authors, and last authors were lower than those of male authors, which is consistent with findings from an Oxford study on the diversity of biomedical research in these authorship categories (Shah et al., 2021; Giannos et al., 2023). This finding is also consistent with those from other Arab countries (Albahari and Bashir, 2020b,a; Rachid et al., 2021). In Bahrain, the mean proportion of female Bahraini authors was 36.0%, fluctuating over the years but showing no significant closure of the gap, with the lowest proportion in 2010 and a modest peak in 2018. A recent review of female authorship in toxicology journals reported a relatively close percentage (32%) of first authors (Love et al., 2024), and progress in narrowing the gap has been reported in medical education journals (Madden et al., 2021). These disparities extend to journal quality and collaboration types, where male first authors are more frequently represented in high-impact Q1 journals, are part of larger research teams, and are engaged in more international collaborations. The low percentage (3.4%) of journals in our dataset that were not indexed in the SJR database is unlikely to have underestimated our gender pattern. Such trends may reflect underlying factors, such as family obligations, resource allocation biases, and workplace cultures, that disproportionately affect female researchers, as noted in the broader literature on gender inequities in academia (Ismail et al., 2013; Lerchenmuller et al., 2018; Mairesse and Pezzoni, 2015). The female authors were mainly original articles across senior authorship positions (83.3%−87.9%). An Indian study on authorship in oncology research reported that original articles were predominant (30.9%) among female authors (Noronha et al., 2024).

Gender disparities in medical authorship have been observed in various low- and middle-income countries, extending beyond the Arab region. A recent cross-sectional analysis of 6,088 articles from 54 Colombian medical journals (2018–2022) revealed that women comprised approximately 46% of first authors and 41% of last authors in original research articles, with even lower representation in surgical specialties such as orthopedics (10%) and neurosurgery (27%), along with geographic variations (Gutiérrez Torres et al., 2025). These findings are consistent with our research in Bahrain, underscoring the necessity for region-specific interventions to promote equitable authorship opportunities for female researchers in academic medicine. Beyond the statistically significant gender disparity observed in publication counts and senior authorship in our study, a more nuanced interpretation necessitates integrating our quantitative findings with established structural and cultural barriers to female academic advancement. The underrepresentation of female authors in Q1 journals likely reflects challenges in securing highly competitive research funding, a prerequisite for large-scale, high-impact projects. Although institutional policies are designed to ensure equitable access to grants, constraints on time and resources—particularly those arising from greater family and caregiving responsibilities—may impact female researchers' propensity to apply for complex funding and their capacity to lead resource-intensive projects. The observed collaboration patterns further evidence this. The reduced frequency of international collaboration among female researchers can be attributed to lower attendance at global conferences and fewer opportunities for international networking, often resulting from challenges related to work-life balance. Conversely, the higher rates of institutional and national collaboration among women may reflect a pragmatic response to these limitations, prioritizing locally accessible and manageable research partnerships. Additionally, the finding that female corresponding authors include a significantly higher proportion of female coauthors suggests the formation of gender-congruent supportive networks. In contexts where cultural norms may influence cross-gender professional interactions, these networks serve as vital support systems, fostering a collaborative environment perceived as more comfortable and practical. Ultimately, while this study clearly delineates the quantitative gaps, a comprehensive understanding of their origins—including the influence of workplace culture, resource allocation biases, and specific career interruptions—will require subsequent qualitative studies.

Despite the observed gaps, encouraging signs emerge from the data, including a fivefold increase in overall medical and biomedical publications from 2005 to 2024, primarily driven by institutions such as AGU, which led in authorship across various fields. This recent advancement in gender parity is in line with what has been reported in plastic surgery research (Jeong et al., 2025). The corresponding female authors demonstrated a stronger inclination toward including female Bahraini coauthors. They focused more on local collaborations, potentially indicating efforts to foster inclusive networks within Bahrain's academic and health sectors. This finding is similar to what has been reported in the literature (Jeong et al., 2025). However, the concentration of publications in local and regional journals, combined with a low international collaboration rate compared with other GCC countries (Ranjan et al., 2016) highlights Bahrain's challenges in achieving global research visibility. Low international collaboration may limit Bahraini scholars' ability to achieve global standing (Gutiérrez Torres et al., 2025). It also affects networking and the quality of publications and can delay the promotion of academic faculty, as publishing in Q1 journals has become an advantage for promotion.

To address these disparities, targeted interventions such as workshops on research methodology, English editing services, mentorship programs, equitable funding distribution, and policies promoting work–life balance could empower female researchers, ultimately enhancing Bahrain's medical and biomedical output and contributing to regional gender equity in science. Future studies should explore qualitative factors, including barriers specific to Bahraini women, to inform more nuanced strategies. Finally, although this is a case study of Bahrain, we encourage other Arab countries to replicate this study and compare their findings with ours to gain a broader understanding of the gender gap among Arab medical and biomedical researchers and propose solutions to narrow it.

### 4.1 Strengths and limitations

This study presents a comprehensive analysis of gender disparities in medical and biomedical research productivity in Bahrain over 20 years (2005–2024), using a robust bibliometric approach based on the Scopus database. The inclusion of a wide range of variables, such as authorship positions, journal quartiles, SJR, and collaboration types, strengthens the depth and granularity of the findings. The study's focus on Bahrain-specific institutions, including those in the academic and health sectors, uniquely contributes to the limited literature on gender differences in research productivity in the Arab region. By identifying key institutions, such as AGU, as leaders in publication output and analyzing trends over time, this study provides actionable insights for policymakers and academic leaders seeking to promote gender equity. Additionally, the rigorous methodology, including the use of statistical tests and the exclusion of duplicate entries, enhances the reliability and validity of the results.

Despite its strengths, the study has several limitations. First, reliance on the Scopus database may have excluded publications not indexed in that database. Although Scopus provides the most comprehensive and standardized coverage of Bahraini medical and biomedical publications, including the three leading local journals that account for the majority of national output, a small proportion of articles published in non-indexed local periodicals or conference proceedings may have been missed. This could marginally underestimate the total publication volume, although it is unlikely to substantially alter the observed gender patterns given the dominance of Scopus-indexed journals in the dataset. Second, in limited instances, determining the names of Arab authors who are gender-neutral or culturally ambiguous, as well as those of non-Arab authors, may have introduced slight inaccuracies through searches of their institutions' websites and other online sources. External verification was required for fewer than 1% of author occurrences and was conducted using multiple corroborating sources. We acknowledge that a minimal degree of residual gender misclassification cannot be completely ruled out for authors with limited or contradictory online profiles. However, given the low proportion and the conservative verification protocol applied, any such error is unlikely to affect the overall patterns or statistical conclusions reported. Third, excluding the number of authors and institutions above 15 from the calculation of the average number of authors and institutions might have slightly underestimated the numbers. Fourth, the author's first affiliation may have impacted the representation of some institutions. Fifth, the study did not account for qualitative factors such as career interruptions, family responsibilities, or institutional barriers, which could provide a deeper context for the observed gender disparities.

## 5 Conclusions

This study highlights a significant gender disparity in the productivity of medical and biomedical research output in Bahrain from 2005 to 2024, with male authors from Bahrain consistently outnumbering females across first, last, and corresponding authorship positions. Despite a fivefold increase in publication output over the study period, the gender gap persists, with females constituting only 36.0% of Bahraini authors on average and lower representation in high-impact Q1 journals and international collaborations. AGU emerged as the leading institution, contributing significantly to Bahrain's medical and biomedical research output, yet female researchers face challenges in achieving equitable representation, particularly in senior authorship roles. However, female corresponding authors showed a notable tendency to include more female Bahraini coauthors, suggesting efforts toward fostering inclusive local research networks. These findings underscore the need for targeted interventions, such as mentorship, equitable funding, and policies that address work–life balance, to close the gender gap and enhance Bahrain's research ecosystem. Future research should explore qualitative barriers and extend the analysis to other Arab countries to contextualize these disparities within the broader region.

## Data Availability

The original contributions presented in the study are included in the article/Supplementary material, further inquiries can be directed to the corresponding author.
