# Supplementary material for: Medical and biomedical research productivity in Bahrain: an analysis of gender differences and patterns over two decades
Source: Front Res Metr Anal. 2026 Feb 4;11:1714436. doi: 10.3389/frma.2026.1714436 (PMC12913561; doi:10.3389/frma.2026.1714436)
Supplement: Supplementary file 1 [file Table_1.docx]

**Supplementary Table 1.** Journals with the highest proportion of publications by nationality of first, last, and corresponding authors

| **Journal** | | **First Author** | | | | **Last Author** | | | | **Corresponding Author** | | | |
| --- | --- | --- | --- | --- | --- | --- | --- | --- | --- | --- | --- | --- | --- |
|  |  | **Bahraini** | | **Non-Bahraini** | | **Bahraini** | | **Non-Bahraini** | | **Bahraini** | | **Non-Bahraini** | |
|  |  | **n** | **%** | **n** | **%** | **n** | **%** | **n** | **%** | **n** | **%** | **n** | **%** |
| 1 | Bahrain Medical Bulletin (n=808) | 790 | 97.8 | 18 | 2.2 | 610 | 75.5 | 198 | 24.5 | 734 | 90.8 | 74 | 9.2 |
| 2 | Journal of the Bahrain Medical Society (n=148) | 142 | 95.9 | 6 | 4.1 | 104 | 70.3 | 44 | 29.7 | 121 | 81.8 | 27 | 18.2 |
| 3 | Saudi Medical Journal (n=66) | 55 | 83.3 | 11 | 16.7 | 36 | 54.5 | 30 | 45.5 | 54 | 81.8 | 12 | 18.2 |
| 4 | The Lancet (n=58) | 0 | 0.0 | 58 | 100.0 | 0 | 0.0 | 58 | 100.0 | 0 | 0.0 | 58 | 100.0 |
| 5 | PLoS ONE (n=50) | 17 | 34.0 | 33 | 66.0 | 14 | 28.0 | 36 | 72.0 | 18 | 36.0 | 32 | 64.0 |
| 6 | Arab Gulf Journal of Scientific Research (n=46) | 40 | 87.0 | 6 | 13.0 | 33 | 71.7 | 13 | 28.3 | 39 | 84.8 | 7 | 15.2 |
| 7 | Cochrane Database of Systematic Reviews (n=46) | 20 | 43.5 | 26 | 56.5 | 15 | 32.6 | 31 | 67.4 | 14 | 30.4 | 32 | 69.6 |
| 8 | BMJ Case Reports (n=44) | 35 | 79.5 | 9 | 20.5 | 31 | 70.5 | 13 | 29.5 | 34 | 77.3 | 10 | 22.7 |
| 9 | Nutrients (n=41) | 16 | 39.0 | 25 | 61.0 | 12 | 29.3 | 29 | 70.7 | 13 | 31.7 | 28 | 68.3 |
| 10 | Scientific Reports (n=39) | 11 | 28.2 | 28 | 71.8 | 12 | 30.8 | 27 | 69.2 | 11 | 28.2 | 28 | 71.8 |
| 11 | Eastern Mediterranean Health Journal (n=36) | 29 | 80.6 | 7 | 19.4 | 24 | 66.7 | 12 | 33.3 | 30 | 83.3 | 6 | 16.7 |
| 12 | International Journal of Surgery Case Reports (n=28) | 23 | 82.1 | 5 | 17.9 | 20 | 71.4 | 8 | 28.6 | 21 | 75.0 | 7 | 25.0 |
| 13 | International Journal of Molecular Sciences (n=26) | 15 | 57.7 | 11 | 42.3 | 18 | 69.2 | 8 | 30.8 | 18 | 69.2 | 8 | 30.8 |
| 14 | British Journal of General Practice (n=26) | 22 | 84.6 | 4 | 15.4 | 0 | 0.0 | 26 | 100.0 | 22 | 84.6 | 4 | 15.4 |
| 15 | Frontiers in Endocrinology (n=25) | 7 | 28.0 | 18 | 72.0 | 12 | 48.0 | 13 | 52.0 | 10 | 40.0 | 15 | 60.0 |
| 16 | Angiology (n=25) | 0 | 0.0 | 25 | 100.0 | 3 | 12.0 | 22 | 88.0 | 1 | 4.0 | 24 | 96.0 |
| 17 | Oman Medical Journal (n=24) | 17 | 70.8 | 7 | 29.2 | 16 | 66.7 | 8 | 33.3 | 17 | 70.8 | 7 | 29.2 |
| 18 | Diabetes Research and Clinical Practice (n=23) | 7 | 30.4 | 16 | 69.6 | 12 | 52.2 | 11 | 47.8 | 14 | 60.9 | 9 | 39.1 |
| 19 | Journal of Infection and Public Health (n=21) | 13 | 61.9 | 8 | 38.1 | 12 | 57.1 | 9 | 42.9 | 11 | 52.4 | 10 | 47.6 |
| 20 | Annals of Saudi Medicine (n=21) | 14 | 66.7 | 7 | 33.3 | 15 | 71.4 | 6 | 28.6 | 15 | 71.4 | 6 | 28.6 |
| 21 | World Journal of Clinical Pediatrics (n=21) | 18 | 85.7 | 3 | 14.3 | 14 | 66.7 | 7 | 33.3 | 20 | 95.2 | 1 | 4.8 |
| 22 | Sultan Qaboos University Medical Journal (n=20) | 18 | 90.0 | 2 | 10.0 | 16 | 80.0 | 4 | 20.0 | 17 | 85.0 | 3 | 15.0 |
| 23 | BMJ Open (n=20) | 3 | 15.0 | 17 | 85.0 | 5 | 25.0 | 15 | 75.0 | 4 | 20.0 | 16 | 80.0 |
| 24 | Radiology Case Reports (n=20) | 20 | 100.0 | 0 | 0.0 | 19 | 95.0 | 1 | 5.0 | 20 | 100.0 | 0 | 0.0 |
| 25 | All other journals (n=3765) | 1799 | 47.8 | 1966 | 52.2 | 1453 | 38.6 | 2312 | 61.4 | 1758 | 46.7 | 2007 | 53.3 |
