# Supplementary material for: Medical and biomedical research productivity in Bahrain: an analysis of gender differences and patterns over two decades
Source: Front Res Metr Anal. 2026 Feb 4;11:1714436. doi: 10.3389/frma.2026.1714436 (PMC12913561; doi:10.3389/frma.2026.1714436)
Supplement: Supplementary file 2 [file Table_2.docx]

**Supplementary Table 2.** Distribution of documents published in journals according to the gender of Bahraini first, last, and corresponding authors

| **Journal** | | **First Author** | | | | **Last Author** | | | | **Corresponding Author** | | | |
| --- | --- | --- | --- | --- | --- | --- | --- | --- | --- | --- | --- | --- | --- |
|  |  | **Male** | | **Female** | | **Male** | | **Female** | | **Male** | | **Female** | |
|  |  | **n** | **%** | **n** | **%** | **n** | **%** | **n** | **%** | **n** | **%** | **n** | **%** |
| 1 | Bahrain Medical Bulletin (n=808) | 397 | 21.4 | 393 | 30.9 | 360 | 21.3 | 250 | 30.7 | 374 | 18.9 | 360 | 34.7 |
| 2 | Journal of the Bahrain Medical Society (n=148) | 81 | 4.4 | 61 | 4.8 | 70 | 4.1 | 34 | 4.2 | 70 | 3.5 | 51 | 4.9 |
| 3 | Saudi Medical Journal (n=66) | 32 | 1.7 | 23 | 1.8 | 19 | 1.1 | 17 | 2.1 | 35 | 1.8 | 19 | 1.8 |
| 4 | The Lancet (n=58) | 0 | 0.0 | 0 | 0.0 | 0 | 0.0 | 0 | 0.0 | 0 | 0.0 | 0 | 0.0 |
| 5 | PLoS ONE (n=50) | 6 | 0.3 | 11 | 0.9 | 10 | 0.6 | 4 | 0.5 | 9 | 0.5 | 9 | 0.9 |
| 6 | Arab Gulf Journal of Scientific Research (n=46) | 22 | 1.2 | 18 | 1.4 | 27 | 1.6 | 6 | 0.7 | 25 | 1.3 | 14 | 1.4 |
| 7 | Cochrane Database of Systematic Reviews (n=46) | 11 | 0.6 | 9 | 0.7 | 1 | 0.1 | 14 | 1.7 | 6 | 0.3 | 8 | 0.8 |
| 8 | BMJ Case Reports (n=44) | 19 | 1.0 | 16 | 1.3 | 19 | 1.1 | 12 | 1.5 | 21 | 1.1 | 13 | 1.3 |
| 9 | Nutrients (n=41) | 11 | 0.6 | 5 | 0.4 | 9 | 0.5 | 3 | 0.4 | 9 | 0.5 | 4 | 0.4 |
| 10 | Scientific Reports (n=39) | 5 | 0.3 | 6 | 0.5 | 8 | 0.5 | 4 | 0.5 | 4 | 0.2 | 7 | 0.7 |
| 11 | Eastern Mediterranean Health Journal (n=36) | 14 | 0.8 | 15 | 1.2 | 12 | 0.7 | 12 | 1.5 | 15 | 0.8 | 15 | 1.4 |
| 12 | International Journal of Surgery Case Reports (n=28) | 15 | 0.8 | 8 | 0.6 | 19 | 1.1 | 1 | 0.1 | 17 | 0.9 | 4 | 0.4 |
| 13 | International Journal of Molecular Sciences (n=26) | 7 | 0.4 | 8 | 0.6 | 10 | 0.6 | 8 | 1.0 | 5 | 0.3 | 13 | 1.3 |
| 14 | British Journal of General Practice (n=26) | 22 | 1.2 | 0 | 0.0 | 0 | 0.0 | 0 | 0.0 | 22 | 1.1 | 0 | 0.0 |
| 15 | Frontiers in Endocrinology (n=25) | 2 | 0.1 | 5 | 0.4 | 11 | 0.7 | 1 | 0.1 | 4 | 0.2 | 6 | 0.6 |
| 16 | Angiology (n=25) | 0 | 0.0 | 0 | 0.0 | 3 | 0.2 | 0 | 0.0 | 1 | 0.1 | 0 | 0.0 |
| 17 | Oman Medical Journal (n=24) | 11 | 0.6 | 6 | 0.5 | 6 | 0.4 | 10 | 1.2 | 14 | 0.7 | 3 | 0.3 |
| 18 | Diabetes Research and Clinical Practice (n=23) | 5 | 0.3 | 2 | 0.2 | 9 | 0.5 | 3 | 0.4 | 12 | 0.6 | 2 | 0.2 |
| 19 | Journal of Infection and Public Health (n=21) | 5 | 0.3 | 8 | 0.6 | 8 | 0.5 | 4 | 0.5 | 4 | 0.2 | 7 | 0.7 |
| 20 | Annals of Saudi Medicine (n=21) | 9 | 0.5 | 5 | 0.4 | 7 | 0.4 | 8 | 1.0 | 11 | 0.6 | 4 | 0.4 |
| 21 | World Journal of Clinical Pediatrics (n=21) | 17 | 0.9 | 1 | 0.1 | 6 | 0.4 | 8 | 1.0 | 19 | 1.0 | 1 | 0.1 |
| 22 | Sultan Qaboos University Medical Journal (n=20) | 11 | 0.6 | 7 | 0.5 | 12 | 0.7 | 4 | 0.5 | 12 | 0.6 | 5 | 0.5 |
| 23 | BMJ Open (n=20) | 0 | 0.0 | 3 | 0.2 | 3 | 0.2 | 2 | 0.2 | 1 | 0.1 | 3 | 0.3 |
| 24 | Radiology Case Reports (n=20) | 14 | 0.8 | 6 | 0.5 | 12 | 0.7 | 7 | 0.9 | 12 | 0.6 | 8 | 0.8 |
| 25 | All other journals (n=3765) | 1142 | 61.5 | 657 | 51.6 | 1050 | 62.1 | 403 | 49.4 | 1278 | 64.5 | 480 | 46.3 |
| **Total** | | 1858 | 100.0 | 1273 | 100.0 | 1691 | 100.0 | 815 | 100.0 | 1980 | 100.0 | 1036 | 100.0 |
